# Investigating Substitutions in Antibody–Antigen Complexes Using Molecular Dynamics: A Case Study with Broad-spectrum, Influenza A Antibodies

**DOI:** 10.3389/fimmu.2017.00143

**Published:** 2017-02-15

**Authors:** William D. Lees, Lenka Stejskal, David S. Moss, Adrian J. Shepherd

**Affiliations:** ^1^Institute of Structural and Molecular Biology, Birkbeck College, London, UK

**Keywords:** influenza, hemagglutinin, broad-spectrum antibodies, molecular dynamics, MM/GBSA

## Abstract

In studying the binding of host antibodies to the surface antigens of pathogens, the structural and functional characterization of antibody–antigen complexes by X-ray crystallography and binding assay is important. However, the characterization requires experiments that are typically time consuming and expensive: thus, many antibody–antigen complexes are under-characterized. For vaccine development and disease surveillance, it is often vital to assess the impact of amino acid substitutions on antibody binding. For example, are there antibody substitutions capable of improving binding without a loss of breadth, or antigen substitutions that lead to antigenic escape? The questions cannot be answered reliably from sequence variation alone, exhaustive substitution assays are usually impractical, and alanine scans provide at best an incomplete identification of the critical residue–residue interactions. Here, we show that, given an initial structure of an antibody bound to an antigen, molecular dynamics simulations using the energy method molecular mechanics with Generalized Born surface area (MM/GBSA) can model the impact of single amino acid substitutions on antibody–antigen binding energy. We apply the technique to three broad-spectrum antibodies to influenza A hemagglutinin and examine both previously characterized and novel variant strains observed in the human population that may give rise to antigenic escape. We find that in some cases the impact of a substitution is local, while in others it causes a reorientation of the antibody with wide-ranging impact on residue–residue interactions: this explains, in part, why the change in chemical properties of a residue can be, on its own, a poor predictor of overall change in binding energy. Our estimates are in good agreement with experimental results—indeed, they approximate the degree of agreement between different experimental techniques. Simulations were performed on commodity computer hardware; hence, this approach has the potential to be widely adopted by those undertaking infectious disease research. Novel aspects of this research include the application of MM/GBSA to investigate binding between broadly binding antibodies and a viral glycoprotein; the development of an approach for visualizing substrate–ligand interactions; and the use of experimental assay data to rescale our predictions, allowing us to make inferences about absolute, as well as relative, changes in binding energy.

## Introduction

The identification and characterization of broadly neutralizing antibodies (bnAbs) to highly mutable pathogens such as HIV (1) and influenza (2, 3) has important consequences both for treatment and for vaccine development, but the structural understanding of antibody/antigen interactions is far from complete. An important area of focus is the impact on antibody binding of amino acid substitutions in the epitope—could mutation lead to escape?—or in the paratope—could binding be improved, without loss of breadth? Today, key residues are typically identified by means of alanine substitution assays, which determine ΔΔ*G*_bind_, the change in the Gibbs free energy of binding caused by the substitution. These assays can be expensive and time consuming to conduct, particularly if the required antibody is not to hand, and can only provide a limited understanding of the contribution that individual residues make to the interaction. In particular, where exhaustive scans are conducted over the entire epitope, the sum of per-residue free-energy contributions inferred from alanine scanning is found to differ significantly from the wild-type (WT) free energy (4). Residues are therefore found to bind cooperatively, meaning that the contribution of individual residues is non-additive. Additionally, substitution assays cannot reliably identify the extent of intramolecular contacts. In particular, there are known examples of substitutions outside such contacts that significantly affect ΔΔ*G*_bind_, and examples of contact residues that are not identified by alanine substitution (5). Thus, a crystal structure of the complex remains necessary.

In this report, we study the binding characteristics and sensitivity to mutations of three bnAbs to influenza for which structures and binding assays exist. We demonstrate that molecular dynamics (MD) and other computational techniques, running on widely available computer hardware, can be used to complement experimental results in order to gain a deeper insight into molecular interactions and can also predict the structural and chemical impact of substitutions.

Broadly neutralizing antibodies to influenza A hemagglutinin (HA), a surface glycoprotein implicated in host cell attachment, challenge the conventional wisdom that the B-cell response to influenza must always be governed by antigenic drift, and raise the possibility of broad-spectrum therapeutic treatment and of vaccines that could confer longer lasting protection (6). Such bnAbs were initially thought to be rare but have now been isolated in many studies. Examples exist that bind both to the HA globular head and to the stalk. The functional mechanism of stalk-binding bnAbs is largely through inhibition of cell membrane fusion, although other mechanisms such as antibody-dependent cell-mediated cytotoxicity may also play a part (7). While the stalk-binding antibodies characterized to date arise from several germlines, the V_H_1–69 germline is frequently found, and around 40 bnAbs arising from this germline have been described to date. Reported common features of these antibodies include binding through the heavy chain only; distinctive hydrophobic substitutions in HCDR2 and tyrosine(s) in HCDR3 (8).

In this study, we use MD simulations employing the energy method molecular mechanics with Generalized Born surface area (MM/GBSA) in combination with experimental results published previously by other groups. We examine three stalk-binding V_H_1–69 based bnAbs: CR6261, which neutralizes influenza A group 1 strains (3), CR9114, which neutralizes influenza A group 1 and group 2 strains as well as influenza B strains (9), and CR8020, which neutralizes group 2 strains only (10). We compare predictions obtained from MM/GBSA with experimental results, and with a computational service, ANCHOR, which was used in one of the experimental studies, and which, in the examples we study, is found to provide useful initial results for a small computational cost.

Molecular mechanics with Generalized Born surface area is an approximate method for inferring the change in Gibbs free energy, Δ*G*, caused by substrate/ligand binding (11). The method uses a thermodynamic cycle to infer the solvated free energy of association, which we shall refer to for brevity as Δ*G*_bind_. The inference is derived from calculations of the free energy of association in vacuum, Δ*G*_bind,vac_, and of the solvation energy Δ*G*_solv_ of the ligand, receptor, and complex, using the following equation:

$$\text{Δ}G_{\text{bind}}=\text{Δ}G_{\text{bind,vac}}+\text{Δ}G_{\text{solv,complex}}-\text{(Δ}G_{\text{solv,ligand}}+\text{Δ}G_{\text{solv,receptor}}\text{)}$$

The calculation averages results obtained from an ensemble of uncorrelated snapshots, collected from an MD simulation of the explicitly solvated complex and its components. Δ*G*_solv_ is determined by solving the linearized Generalized Born equation (to determine the electrostatic contribution) and by a term to account for enthalpic and entropic hydrophobic contributions: these contributions are typically taken to be linearly proportional to the solvent-exposed surface area and are calibrated with reference to experimentally determined hydration energies of small molecules. As Generalized Born is an implicit solvent model, solvent molecules are removed from the MD snapshot before the calculation of Δ*G*_solv_ is performed: this avoids the otherwise lengthy convergence times that would be required to smooth the fluctuations in solvent–solvent interactions. Δ*G*_bind,vac_ can be expressed in terms of enthalpic and entropic contributions:

$$\text{Δ}G_{\text{bind,vac}}=\text{Δ}E_{\text{bind,vac}}-\text{TΔ}S_{\text{bind,vac}}$$

In this application, we adopt the commonly used approximation that, as the overall conformations of ligand and substrate are not substantially affected by the substitutions tested, the conformational entropy change caused by a substitution, ΔΔ*S*_bind,vac_, is negligible. As we are principally interested in ΔΔ*G* rather than Δ*G*, we do not calculate Δ*S*_bind,vac_. Our inferred values of Δ*G* will therefore not agree with experimentally determined values: however, within the limits of our approximation that ΔΔ*S*_bind,vac_ ≈ 0, determinations of Δ*G*_bind_ may be compared in order to understand relative energies of interaction. Δ*E*_bind,vac_ consists of the molecular mechanics contributions from bonded, electrostatic, and van der Waals interactions, determined from the receptor, ligand, and complex structures elicited from the MD simulation. Receptor, ligand, and complex structures may be determined from independent simulations, or, on the assumption that no significant conformational changes occur during binding, they may be extracted from a single trajectory of the complex. In our experience, the use of independent simulations greatly increases convergence times as frames from the separate simulations are not correlated: we therefore employ a single trajectory in this case.

We use MM/GBSA both to infer the overall value of ΔΔ*G*_bind_ for a given complex, and to determine the pairwise ΔΔ*G*_bind_ between individual residues in the epitope and paratope through a calculation known as energy decomposition. In this calculation, the binding free energy attributable to the interaction between a specific pair of residues is determined, from the Generalized Born equation, as the sum of the electrostatic and hydrophobic terms (the contribution to ΔΔ*G*_bind,vac_ from ΔΔ*S*_bind,vac_, which we assume, as above, to be negligible, cannot be attributed to individual residue pairs and is not included in the calculation). Although it is subject to limits of error imposed by the approximations in the calculation, the determination of these pairwise energies allows an understanding of the interaction that is not available directly from experimental methods. ΔΔ*G*_bind_ predicted by MM/GBSA is typically found to be proportional to but not identical to experimentally determined ΔΔ*G*, likely as a result of inconsistent energy functions in the explicit and implicit solvent terms underlying the MM/GBSA calculation (11–13). As some experimental assays are available for the complexes we study, we use these to rescale our predictions.

Molecular dynamics has provided valuable insights into the structural consequences of substitutions in Ab/influenza HA complexes (14, 15). Molecular mechanics with Poisson–Boltzmann surface area (MM/PBSA) and MM/GBSA have been used in at least one previous study to predict ΔΔ*G*_bind_ in an antibody/HA interaction that corresponded with experimental results (16). In this study, we develop novel interaction energy diagrams to facilitate such analysis and use MD in combination with MM/GBSA to develop insights into the binding characteristics of broad-spectrum stalk-binding antibodies by studying a range of substitutions and Abs. We examine and contrast several substitutions in the epitope and paratope, and we apply current best practice in MM/GBSA calculations by monitoring sample correlation and convergence of results. To our knowledge, this is the first comparative study of broadly binding antibodies to viral glycoprotein using MD.

## Materials and Methods

### Starting Structures

Starting structures, 3GBM (17), 4FQY (9), and 3SDY (10) (resolutions 2.7, 5.25, and 2.85 Å, respectively) were downloaded from the RCSA Protein Data Bank (18). Residues in antibody loops remote from the epitope for which no coordinates were reported were modeled with MODELLER (19). Protonation states consistent with physiological conditions were inferred by MolProbity (20). The trimeric biological construct was built in UCSF Chimera (21), using the author-defined biological unit, and the starting structure for simulation was created in AmberTools 15 *tleap* using the Amber ff12SB force field (22) and mbondi2 radius sets (23). Epitope substitutions were modeled in MODELLER using the single chain output by MolProbity, following which the same steps were taken to create the starting structure for simulation.

### Explicit Solvent Simulations

For explicit solvent simulations, the structure was solvated in a truncated octahedral box using TIP3P water molecules. Na^+^ ions were added to neutralize the molecular charge. A minimum distance of 20 Å was enforced between the structure and the box boundary, leading to boxes of volume 6 × 10^6^ Å^3^ containing a total of ~6 × 10^5^ atoms. Simulations were conducted in Amber 14 using the GPU-based simulation engine (24–26).

Both the WT structure and structures with substitutions underwent an initial energy minimization stage at constant volume, consisting of 2,000 steps of the steepest descent method, followed by 2,000 steps of the conjugate gradient method. This was followed by two relaxation steps: 100 ps at constant volume with backbone atoms restrained by a force equivalent to 4.0 kcal/mol/Å during which the temperature was increased from 0 to 50 K using a Langevin thermostat; then 2000 ps at constant pressure using a Monte Carlo barostat and Langevin thermostat, with backbone atoms restrained by a force equivalent to 1.0 kcal/mol/Å. In this step, the temperature was increased from 50 to 310 K in the first 500 ps and then held at 310 K for the remainder.

Following the relaxation steps, the production run was conducted at constant pressure using a Monte Carlo barostat. The initial production step consisted of a 9 ns simulation and was followed by 20 independent simulations of 1 ns each. These simulations were started with position coordinates from the end of the initial 9 ns simulation, but randomized velocities, in order to de-correlate the output from successive samples (27). A short-range interaction cutoff distance of 8 Å was used throughout, and SHAKE was used to constrain hydrogens in all but the minimization step, allowing a 2 fs time step. *iwrap* was set to 1, wrapping atomic coordinates back into the primary box, in order to avoid coordinate overflow during the course of long simulations. Default values were used for other modeling parameters.

**Figure 1:**
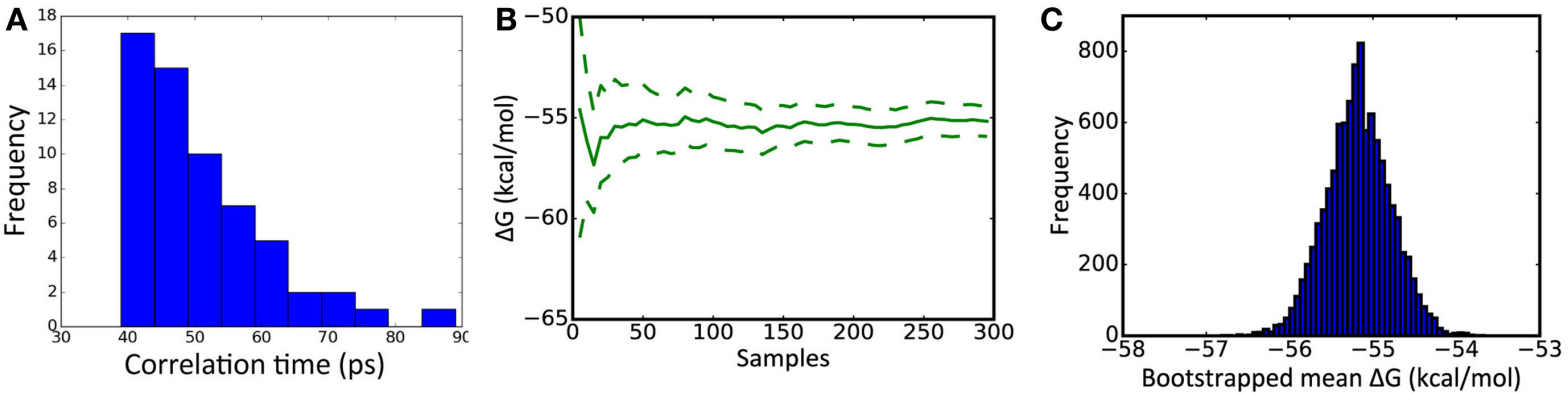
**Key metrics underlying molecular mechanics with Generalized Born surface area (MM/GBSA) calculations**. **(A)** Distribution of the correlation time of MM/GBSA Δ*G* energy estimates, calculated from 20 1 ns simulations yielding 60 snapshots. **(B)** Example cumulative estimate of Δ*G* as the number of samples is increased [dotted lines represent 95% confidence limits (see Analysis of MM/GBSA Calculations)]. **(C)** Example distribution of a mean Δ*G* obtained from 10,000 samplings of the underlying dataset. **(B,C)** are taken from the analysis of substitution CR8020 D19N.

Snapshots were extracted from each of the 20 independent simulations for further calculations. To allow time for velocities to settle, the first snapshot was taken after 500 ps of simulation time. To minimize correlation between snapshots, subsequent snapshots were taken at times 100 ps apart, resulting in a total of five snapshots being extracted from each of the 20 simulations. The sampling interval of 100 ps was determined by examining the time required for the time-dependent autocorrelation function [as determined by the Python function numpy.correlate (28)] to reduce to 0, using MM/GBSA Δ*G* values obtained from more closely sampled snapshots (Figure 1A). The entire simulation of a structure (29 ns of simulation) took approximately 6 days on our Nvidia Titan/X GPU hardware.

### Coordinate Analysis

Analysis of simulation coordinates was performed using *cpptraj* from Amber Tools 15. The 100 snapshots were analyzed for atomic contacts between HA1/HA2 and the Ab variable regions with a distance cutoff of 3.9 Å, sufficient for the identification of hydrogen bonds and other hydrophobic contacts. All corresponding residues were added to the list of potential contact residues regardless of the number of snapshots in which the contact was observed. The approach taken here is inclusive of the molecular conformations observed during the simulation. Hydrogen bonds were inferred between hydrogen atoms bonded to nitrogen or oxygen atoms, where the donor and acceptor heavy atoms concerned were less than 3.35 Å apart, and the acceptor-hydrogen-donor angle is greater than 135°. The hydrogen bond analysis was run over the same set of snapshots as for contact residues.

### MM/GBSA Calculations

Energy calculations were conducted using MMPBSA.py from Amber Tools 15 (29). In accordance with typical practice (27), configurations in the bound and unbound states were assumed to be similar, and based on this assumption, unsolvated topology files for the bound and unbound species were prepared from the explicit solvent topology using ante-MMPBSA.py. The antibody bound to each monomer was treated independently, meaning that the set of 100 snapshots resulted in a total of 300 samples for implicit solvent simulations. These simulations were run in Amber 14’s *sander* under the control of MMPBSA.py using the Generalized Born model with *igb* = 2 (23). In this model, the non-polar contribution to Δ*G* is calculated as 0.005 kcal/mol for each 1 Å^2^ of solvent-accessible surface area. Salt concentration was set to 0.2 M, and other parameters followed Amber’s defaults. Pairwise free-energy decompositions (*idecomp* = 3) were obtained for all potential contact residues identified in the coordinate analysis. MM/GBSA analysis of a structure took approximately 6 h on a 20-core 2.5 GHz Intel Xeon E5-based server.

### Analysis of MM/GBSA Calculations

The 100 Δ*G*_bind_ values obtained for each of the three antibodies bound to the trimer were obtained *via* MMPBSA.py’s Python API and interleaved to form a single set of 300 results. Convergence was assessed by plotting the mean value obtained after from 10, 20, …, 300 results (Figure 1B). To form an unbiased estimate of the confidence limits, the resulting dataset was resampled 10,000 times: the confidence limits represent the range covered by 95% of the resulting means (Figure 1C).

As noted in the introduction, ΔΔ*G*_bind_ predicted by MM/GBSA is typically found to be proportional to but not identical to experimentally determined ΔΔ*G*. Predicted values from each simulation were therefore plotted against experimental values (Figure S1 in Supplementary Material). The slopes of the lines were used to rescale the predictions.

### ANCHOR Calculations

Structure files were uploaded to the ANCHOR server at http://structure.pitt.edu/anchor. For Ab heavy/light chain residue predictions, Protein 1 was set to the Ab chain, and Protein 2 to each of the two cognate HA chains in turn: the results were then summed. For HA residue predictions of CR9114 and CR6261, Protein 1 was set to either HA1 or HA2, and Protein 2 to the Ab heavy chain. For CR8020, results for both Ab chains were summed.

### Influenza Sequence Variation

The sequence variation (SNP) module of the Influenza Research Database (30) was used to identify the variation of human HA subtypes H1, H3, and H5 (approximately 18,000, 13,000, and 450 strains, respectively) at key contact locations identified in this study. Precise numbers of strains compared varied from residue location to location depending on the extent of available sequences.

### Software

The software underlying our methods is publicly available at https://github.com/williamdlees/AmberUtils (doi:10.5281/zenodo.159170). The software streamlines the handling of substitutions; performs convergence limit calculations; and produces interaction diagrams as used in this report.

## Results

### Epitope and Paratope Determination

**Figure 2:**
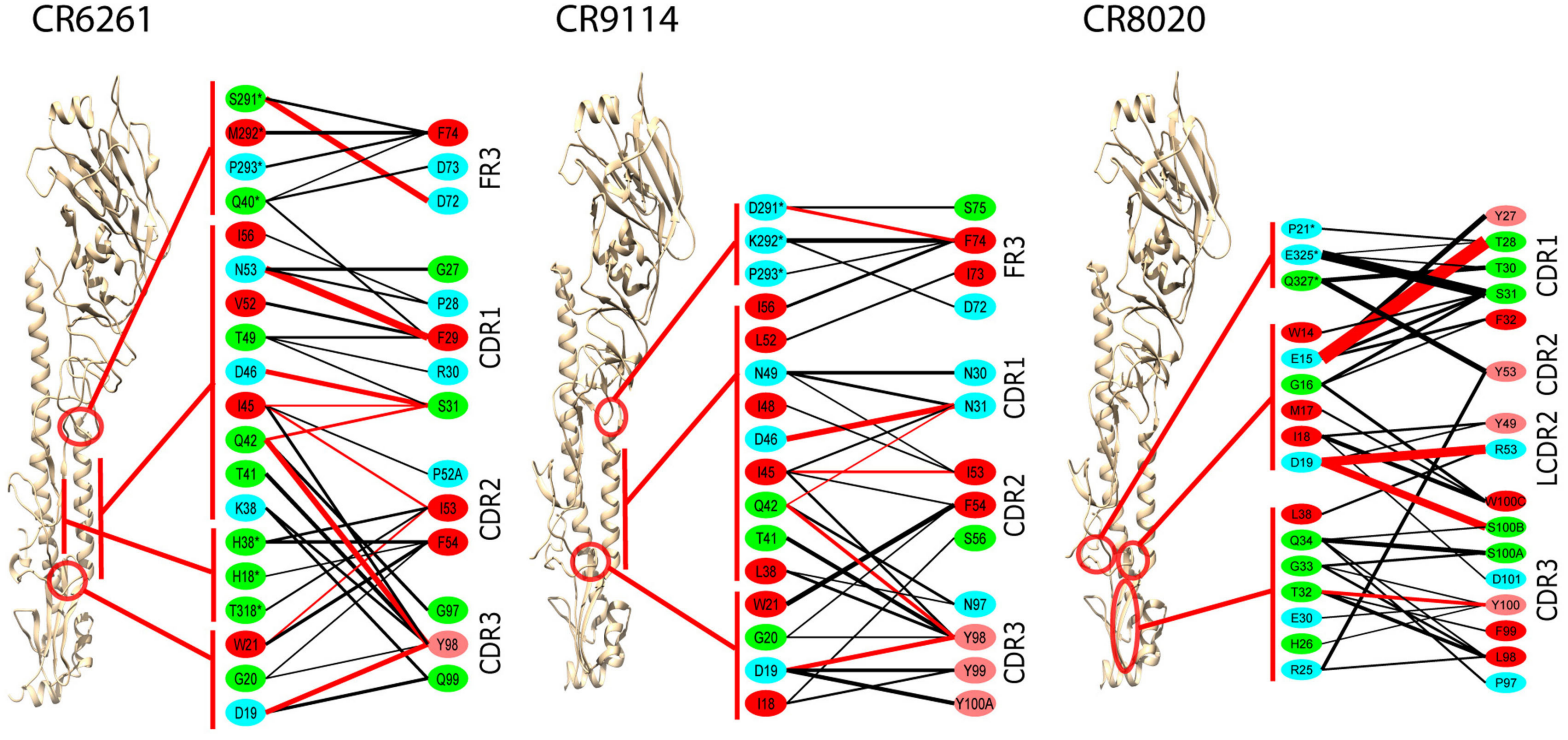
**Averaged interactions between contact residues**. Inferred hydrogen bonds are in red, other interactions in black. Line thickness is proportional to interaction energy. HA1 residues are indicated by *. Residue coloring reflects hydrophobicity at pH 7: red (pink) highly (slightly) hydrophobic, green neutral, blue hydrophilic. To ensure significance, and bearing in mind the overall confidence limits, only interactions with Δ*G* ≤ −1.0 kcal/mol are shown, and residues with no such interactions are omitted. All CDRs are in the heavy chain, except for LCDR2 in the CR8020 interface, which is explicitly marked.

Epitopes and paratopes were inferred from contact residues (Figure 2). The epitopes of CR6261 and CR9114 cover similar regions, although that of CR6261 includes an additional region at the C- and N-termini of HA1. This region includes HA1 38, glycosylated in group 2 strains, which may account for its omission from the CR9114 epitope (9). Interactions with HA1 residues play a stronger part in the CR6261 complex than that of CR9114, with HA1 S291 forming a strong hydrogen bond to HFR3 D72 (predicted Δ*G*_bind_ = −2.7 kcal/mol). In the CR9114 complex, interactions with the three tyrosines at CDR3 98–100 A provide stronger bonds to the N-terminus of HA2 than the single Y98 in CR6261. CR8020 binds closer to the viral membrane than the other two Abs. The HFR3 loop of CR8020 is not in contact with the antigen, but contacts are found in LCDR2 as well as the three HCDRs.

In CR6261 and CR9114, MM/GBSA calculations predict binding energy in the paratope to be evenly spread between four regions of contact: the three HCDRs, and a loop in HFR3 with the tip at locations 72–75 (Table 1). In the epitopes, residues in Helix A account for ~50% of the predicted binding energy: other regions in HA2 account for a further 30%, and ~20% is accounted for by residues in HA1 at the membrane-distal end of the globular head. In CR8020, predicted binding energy is less evenly spread between the CDRs than in the other two complexes, with HCDR1 and HCDR3 accounting for 80% of the binding energy between them. In the epitope, approximately 50% of the predicted binding energy is accounted for by HA2 residues in a β-strand running between the membrane interface and Helix A.

**Table 1:** **Percentage of the total Ab/epitope interaction energy attributable to the individual regions identified in the text**.

Region	% interaction energy		
	3GBM (CR6261,H5)	4FQY (CR9114,H3)	3SDY (CR8020,H3)
HCDR1	34	19	41
HCDR2	19	19	7
HFR3	18	22	0
HCDR3	29	40	39
LCDR2	0	0	13
HA1 head/stem interface	20	16	0
HA2 A helix	55	55	0
HA2 N-terminus	9	29	51
HA1 C- and N-termini	16	0	20
HA2 proximal β-sheet	0	0	29

### Comparison of Predicted ΔΔ*G*_bind_ with Experimental Results

**Figure 3:**
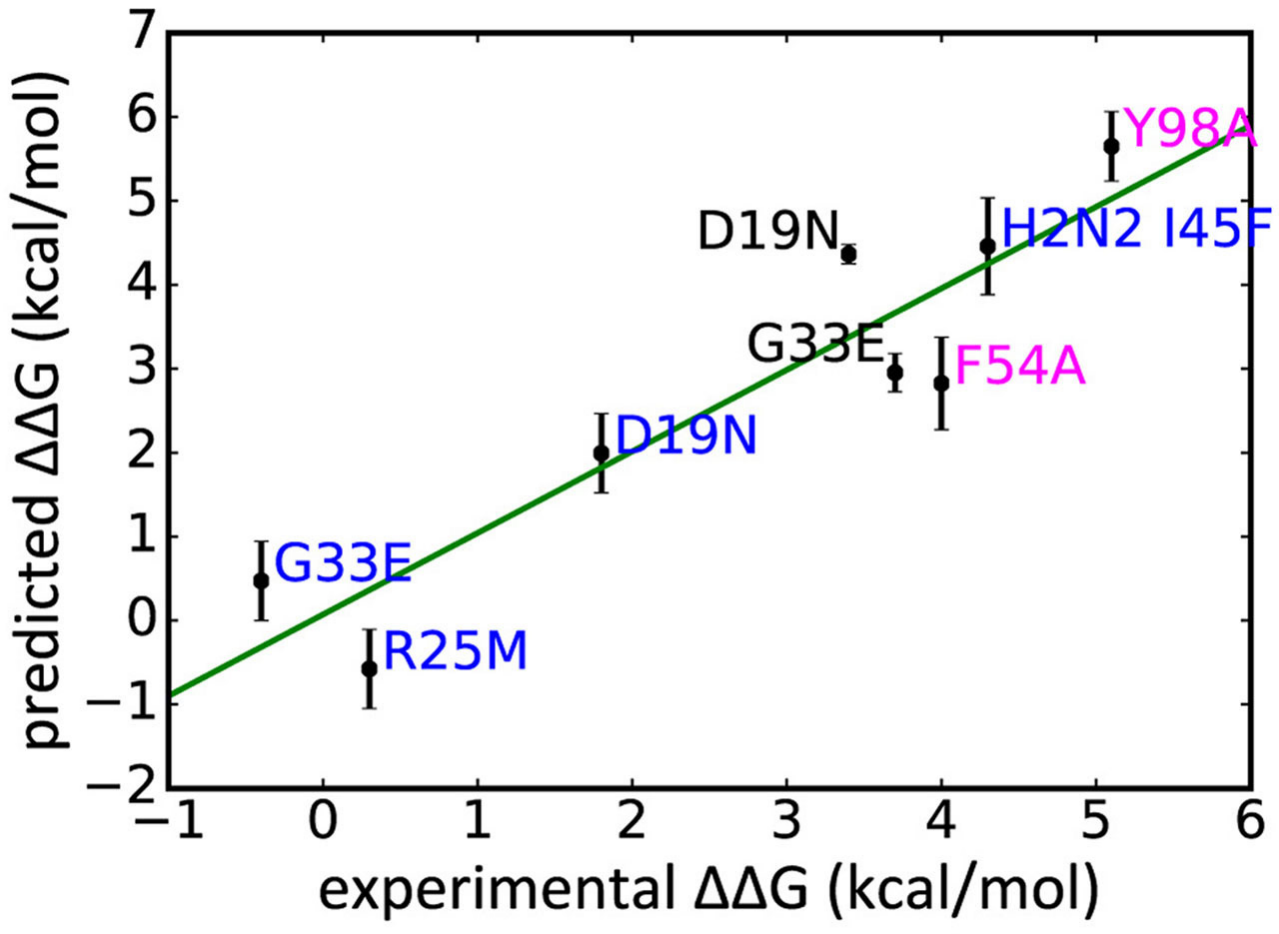
**Experimentally derived ΔΔ*G*_bind_ for a range of substitutions compared with rescaled values predicted in this study, legend colored by Ab: CR9114 blue, CR6261 magenta, CR8020 black**.

In addition to WT assays, we selected seven published assays in which substitutions were applied to the Ab or the epitope (Table 2). These were taken from three different studies: Avnir et al. (8) used the ANCHOR server to predict key residues in the CR6261 HCDRs before verifying results experimentally; Ekiert et al. (10) grew the virus in culture with CR8020 to generate escape mutants, while Dreyfus et al. (9) undertook an extensive panel of substitution assays to examine the impact of variants seen in the wild. In reaching this total of seven substitutions, for reasons of economy and to obtain a spread of results, we excluded a further nine assays of CR9114/H3N2 substitutions from Dreyfus et al. where ΔΔ*G*_bind_ was determined to be <1 kcal/mol, below the 95% confidence limits obtained in this study. We included one substitution, which was assayed in an H2 strain. Using MM/GBSA, we predicted Δ*G*_bind_ for each substitution, and hence ΔΔ*G*_bind_ by comparing with the WT result. Experimental and predicted results are reasonably correlated (Table 2; Figure 3; Figure S1 in Supplementary Material). Error bars reflect the confidence limits in the MM/GBSA calculation, but do not reflect other sources of error such as those introduced by the approximations in our approach and underlying inaccuracies in the force fields. The mean difference between predicted and experimental ΔΔ*G*_bind_ was 0.6 kcal/mol, σ = 0.4 kcal/mol, hence in this application, we find that the technique can predict ΔΔ*G*_bind_ within approximately 1 kcal/mol.

**Table 2:** **Predictions of ΔΔ*G*_bind_ (relative to WT) for substitutions for which experimental values were found**.

Complex	Region	Substitution	ΔΔ*G*_bind_ (kcal/mol)		Source
(Ab, Subtype)			Molecular mechanics with Generalized Born surface area	Experimental	
3GBM (CR6261, H5)	HCDR2	F54A	2.8 ± 0.6	4.0	Avnir et al. (8)
	HCDR3	Y98A	5.6 ± 0.4	5.1	

4FQY (CR9114, H3)	HA2 N-terminus	HA2 D19N	2.0 ± 0.5	1.8	Dreyfus et al. (9)
	A helix	HA2 I45F	4.5 ± 0.6	4.3^a^	
	HA2 proximal β-sheet	HA2 R25M	−0.6 ± 0.5	0.3	
		HA2 G33E	0.5 ± 0.9	−0.4	

3SDY (CR8020, H3)	HA2 N-terminus	HA2 D19N	4.4 ± 0.1	3.4	Ekiert et al. (10)
	HA2 proximal β-sheet	HA2 G33E	3.0 ± 0.2	3.7	

*Ranges for predictions represent bootstrapped 95% confidence limits. Experimental values are derived from published K_d_ values*.

*^a^This assay was conducted against an H2 subtype, as discussed in the text*.

**Figure 4:**
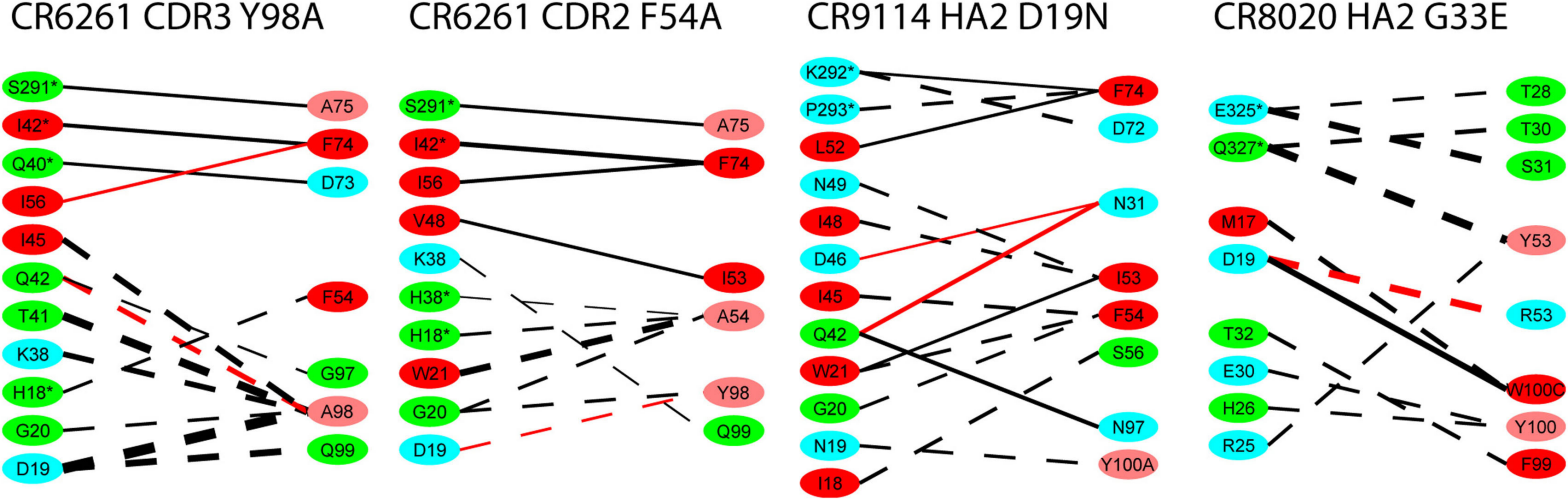
**Interaction energy differences for selected substitutions**. Layout and coloring are the same as for Figure 2, but here the thickness of the line represents the difference in interaction energy relative to the wild type. Dotted lines represent weaker interaction (reduced absolute ΔΔ*G*) and solid lines represent stronger interaction. Only differences with an absolute value ≥1.0 kcal/mol are shown.

**Figure 5:**
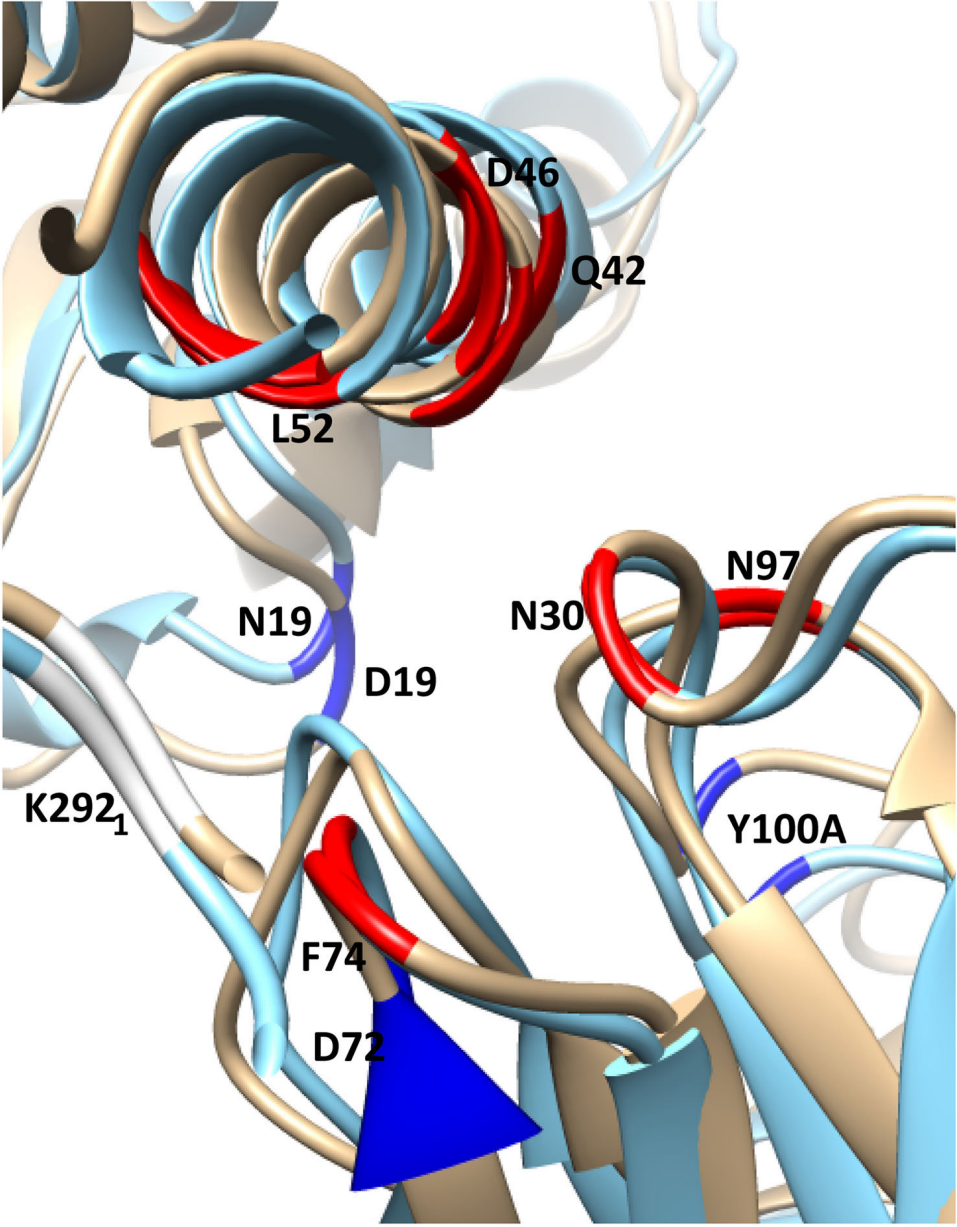
**Selected residues with significant ΔΔ*G*_bind_ in the substitution D19N in the CR9114/H3N2 complex (wild-type chains are in gold, superposed with the substituted complex in blue)**. HA chains are in the top-left corner, Ab chains bottom-right. The structure is viewed along the axis of Helix A, looking toward the viral membrane. The locations of selected residues with stronger interaction in the presence of the substitution are shown in highlighted in red, weaker in dark blue. The weaker interaction between HA2 N19 and CDR3 Y100A allows the antibody’s orientation to shift anticlockwise along the Helix A axis, bringing CDR residues N30, N31, and N97 into closer contact with Helix A residues D46 and Q42. Furthermore, membrane-proximal Ab residues move away from Helix A, while membrane-distal residues move toward it. This latter adjustment weakens the interaction between HA1 K292 and FR3 D72, while strengthening interactions between FR3 F74 and HA1 K292, HA2 L 52.

For the alanine substitutions in the CR6261 HCDRs, ΔΔ*G*_bind_ largely reflects subtraction of the substituted side chain (Figure 4), although compensatory changes to the structure mitigate the overall reduction in binding energy. In some other cases, substitutions with a further reaching impact were observed: in the case of the HA2 substitution D19N in the CR9114 complex, the simulation suggests a change in orientation of the Ab with respect to HA2 Helix A, causing a ripple of stronger and weaker interactions across the interface (Figure 5; Table S2 in Supplementary Material). An equally dispersed reaction was observed in the CR8020 complex with the substitution G33E. Here, a larger side chain is inserted into a congested region of the interface between the proximal region of HA2 and HCDR3. Changes to binding energies and residue distances in the region of the substitution itself were below the limits of error, but, in the MD simulation, the insertion is found to cause a realignment of the Ab that causes distances to other cognate CDRs to increase (Table 3), and this realignment is reflected in predicted pairwise ΔΔ*G*_bind_ (Figure 4).

**Table 3:** **Distances between CA atoms of selected residues, averaged over the final 2 ns of the initial production simulation of the CR8020 complex, with and without the substitution G33E**.

Residues	Distance (Å)	
	Wild type	G33E
HA2 T32–HCDR3 Y100	5.6	5.5
HA1 E325–HCDR1 S31	6.1	9.5
HA1 Q327–HCDR2 Y53	7.0	11.4
HA2 D19–LCDR2 R53	9.9	8.6
HA2 D19–HCDR3 Y100	14.1	13.6

*While the distance between the region in which the substitution is made and its cognate in the HCDR3 is not significantly changed by the substitution, it elicits a re-orientation of the Ab impacting other areas of the interface: in particular those interfacing with HCDR1,2 and LCDR2*.

### Variability of Key Residues in the HA Stalk

We used MM/GBSA analysis to assess substitutions observed in WT strains that we considered likely to have significant impact on Δ*G*_bind_. In key epitope locations identified from pairwise free-energy decompositions, we looked for variants found in at least five sequences deposited at http://fludb.org (Table 4), using the threshold of five as a protection against inaccurate sequencing calls or transcription errors. Notably, we found that HA2 F45 was present in 63 H1 sequences, while HA2 N19 was present in 5 H3 sequences (Table S1 in Supplementary Material). The impact of F45 and N19 on CR9114 binding has been addressed experimentally and by our work above. In addition to the simulations previously described, we simulated the impact of three novel substitutions for which assays have not previously been described: HA2 N49D in the CR9114/H3 complex; HA2 I45F in CR6261/H5; and E325R in CR8020/H3. The predicted ΔΔ*G*_bind_ associated with these three substitutions is comparable to that of experimentally determined escape mutants (Table 5).

**Table 4:** **Wild-type variation at epitope locations found to be key to antibody binding**.

Region	Locations	Epitope	Consensus			Variants		
			H1	H3	H5	H1	H3	H5
HA1 head/stem interface	291	CR6261/CR9114	S	D	S	***N***	***N***EG	–
	292	CR6261/CR9114	L	K	M	FP	***R***NQ	–
HA1 C-terminus	325	CR8020	S	E	Q	FP	RDGK	L
	327	CR8020	Q	Q	E	L	HKP	–
HA2 N-terminus	15	CR8020	T	E	Q	G	–	–
	19	All	D	D	D	N*EL	N*	–
HA2 proximal β-sheet	34	CR8020	Y	Q	Y	–	R	–
HA2 A helix	42	CR6261/CR9114	Q	Q	Q	–	–	–
	45	CR6261/CR9114	I	I	I	NF*V	LTV	–
	49	CR6261/CR9114	T	N	T	QS	D*S***T***	–
	53	CR6261/CR9114	N	N	N	Y	Q	–

*For each complex, the five locations with highest predicted ΔΔ*G*_bind_ were identified for inclusion (some locations being common to more than one complex). For each identified location, the consensus amino acid was determined for the three subtypes shown, as well as variants exhibited by five or more human wild-type strains. Variants in bold italics were covered by assays in Dreyfus et al. (9). Starred variants were modeled in this study*.

**Table 5:** **Substitutions observed in wild-type strains that are predicted from assay and/or calculation to result in significant loss of binding energy**.

Ab	Epitope substitution	Subtype observed in	Tested subtype	ΔΔ*G*_bind_ (kcal/mol)	
				Molecular mechanics with Generalized Born surface area (rescaled)	Experimental
CR6261	I45F	H1 (*n* = 63)	H5	2.4	n.d.
CR9114	D19N	H3 (*n* = 5)	H3	2.0	1.8
	I45F	H1 (*n* = 63)	H3	4.5	4.3^a^
	N49D	H3 (*n* = 5)	H3	1.7	n.d.
CR8020	D19N	H3 (*n* = 5)	H3	4.5	3.4
	E325R	H3 (*n* = 5)	H3	2.8	n.d.

*^a^Conducted against an H2 subtype. The substitutions for which experimental values are available were found to lead to virus escape (9, 10)*.

### Correspondence with ANCHOR Predictions

**Figure 6:**
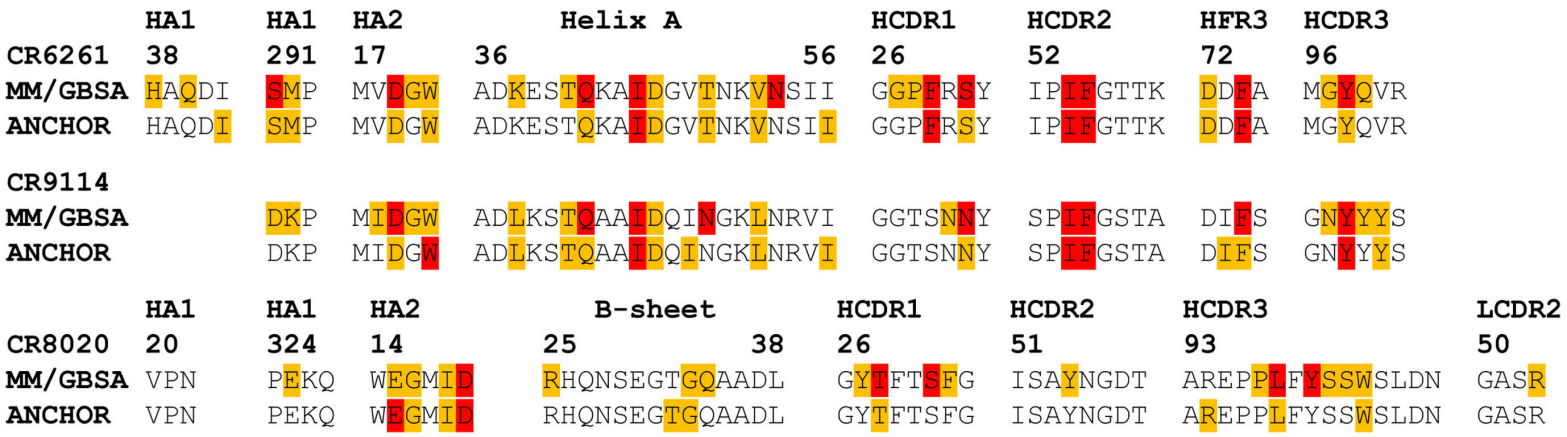
**Comparison of predictions of residue contact free energies from molecular mechanics with Generalized Born surface area (MM/GBSA) with those of ANCHOR**. Following Avnir et al. (8), Anchor predictions ≤−1 kcal/mol and >−3 kcal/mol are shaded amber, those ≤−3 kcal/mol are shaded red. For MM/GBSA, thresholds of −2 and −5 kcal/mol are used for the CR6261 and CR9114 complexes, and −5 and −10 kcal/mol for CR8020: these were found to provide the best correspondence between the two sets of results.

The ANCHOR server at http://structure.pitt.edu/anchor/ provides rapid computational estimates of residue contact free energies, using a combination of Coulombic electrostatic potential and a desolvation term based on interatomic contacts, derived empirically from analysis of protein structures (31). As mentioned previously, Avnir et al. (8) used ANCHOR to identify highly favorable contacts in antibody structures for further examination. We find general correspondence between MM/GBSA and ANCHOR in identifying such contacts (Figure 6), although some highly favorable contacts identified by MM/GBSA are not identified by ANCHOR: in particular, CR6261 CDR3 Y98 is predicted by ANCHOR to be have a lower contact free energy than CDR2 F54 (−2.0 and −3.0 kcal/mol, respectively) although in experimental assays Y98A reduced binding significantly more than F54A (ΔΔ*G*_bind_ −5.1 and −4.0 kcal/mol). In the CR8020 complex, ANCHOR does not predict any highly favorable contacts and predicts a contact free energy >−1 kcal/mol for S31 and Y100, which are predicted by MM/GBSA as highly favorable.

## Discussion

It is now possible to conduct MD simulations of large protein structures on widely available computer hardware, allowing the combinatorial complexity inherent in the study of broad-binding Abs to highly mutable proteins to be explored more widely than would be feasible through experimental techniques alone. Computational techniques can also cast light on the pairwise interactions between residues, which cannot be directly inferred from experimental results. In the experimental work, we cite one study that used surface plasmon resonance (8), while two used biolayer interferometry (9, 10). We have shown that the MM/GBSA analysis can provide predictions of ΔΔ*G*_bind_ for antibodies in complex with influenza HA that are in good agreement with experimental results, and which approximate the accuracy with which biolayer interferometry can match results from surface plasmon resonance assays (32). We have used the technique to identify and explore substitutions of interest for which assay results are not available. The crystal structure of the CR9114 complex had a low resolution (5.7 Å), nevertheless we obtained comparable results: hence, although a structure is required for MM/GBSA analysis, it may in some cases be possible to work with structures of low resolution. The extended MD simulation may have served to refine the structure: alternatively, the low reported resolution may be attributable to flexible regions remote from the interface, such as the unbound light chain.

Molecular mechanics with Generalized Born surface area and MM/PBSA calculations embody approximations over and above those inherent in the MD force field, and they have been found more suited for some applications than for others. Several factors, both in the specific method employed and in its application, may contribute to success in this case. In terms of the method, we monitor convergence and minimize convergence times using independent short simulations. We rescale the predicted energy values to take account of force field inconsistencies inherent in MM/GBSA, using experimentally derived assay values. In terms of the application, the receptor and ligand in this system are large molecules with relatively stable backbone structures, supporting the assumptions that changes in conformational entropy will not be significant and that the system can be appropriately modeled with a single set of simulations rather than with separate simulations for the complex, the receptor and the ligand. Both complex and ligand are pure proteins, meaning that non-protein force fields (which are often not so well developed) are not required. Finally, we simulate single substitutions. Single substitutions are often examined in studies of bnAbs and can provide valuable insights into antibody development and the likelihood of escape; however, benchmarks of energy methods tend to focus on larger-scale substitutions.

The assumption that changes in conformational entropy are negligible remains problematic and may ultimately limit application of the technique. It is helpful in obtaining convergence as the entropy terms in MM/PBSA and MM/GBSA typically have the highest statistical uncertainty (11), but binding enthalpies typically do not correlate well with binding free energies, and the circumstances in which they do are not well understood (33). Changes in entropy can be difficult to determine experimentally, and the significance of entropy/enthalpy compensation is debated (34). Alchemical techniques (35), which maintain explicit solvation throughout and do not require explicit calculation of entropic terms, may ultimately supersede MM/PBSA and MM/GBSA. They are not widely supported on the GPU today, although support in Amber in a forthcoming release has been announced. In current implementations, they do not to our knowledge support per-residue energy decomposition.

In this study, we have not found it necessary to consider the free-energy contribution from ordered water in the interface, which has proved important in some small molecule calculations (11). However, the relatively planar nature of epitopes (36) may make the treatment of explicit water less important than it can be with the more strongly defined cavities typical in such interactions. In identifying contact residues, we have considered hydrogen bonds and other hydrophobic contacts. Longer-range interactions, such as caton–pi interactions, have been identified in some antibody/antigen complexes (37); however, with MM/GBSA, we did not find significant pairwise ΔΔ*G*_bind_ predicted between more distant residues in the complexes studied here.

Having identified key residues in the epitope with MM/GBSA, we were able to identify novel viral mutations observed in the field, that, based on a combination of experimental result and computational prediction, are predicted to lead to the escape of group 1 and group 2 strains from fusogenic inhibition by the bnAbs. We suggest that, when considering the breadth of a bnAb, low-frequency variants should be considered. Of the variants considered in this study, H1 D19N, I45F, and H3 D19N were isolated in multiple geographic locations and across multiple influenza seasons, while H3 N49D strains were isolated from five patients in New York over the course of a single season and H3 E325R was isolated in five patients located in three different cities in Japan over the course of a single season. The diversity of origin, coupled with, in all but one variant, its isolation from multiple subjects in the same city during a single season, suggests that the substitution is viable and hence may have potential to become dominant if there is sufficient antigenic pressure from natural or vaccine-induced Abs (38, 39). Given the potential number of such variants, computational methods such as those presented here may be needed to support such an analysis. Likewise, when assessing vaccine response, to minimize the chances of escape, the depth (i.e., the number of elicited antibodies associated with distinct epitopes or binding patterns) should be considered as well as the breadth.

While in many cases the impact of an epitope or paratope substitution may be relatively local and isolated in effect, in other cases, such as CR9114 HA2 D19N and CR8020 G33E, it can have widespread effects that would be difficult to predict from a list of key residues or understand from a substitution assay result on its own. bnAbs appear to rely less on founder or “anchor” residues than more targeted Abs, having greater evenness of contribution across CDR locations (40). In the case of the structures studied here, particularly CR6261 and CR9114, we can see such evenness of coverage in both the paratope and the epitope. As evenness of coverage increases, so does the likelihood of substitutions having complex, non-local impacts. Likewise, while the overall likelihood of escape may be minimized, the chances of a substitution in the epitope having a significant, albeit non-escaping, impact may increase. For these reasons, a thorough analysis of a broad-spectrum Ab is likely to be more demanding than that of a more highly targeted Ab, and predictions made on the basis of chemical properties or straightforward inferences of critical residues may not be reliable. While the HA stalk experiences substantially less variation than the globular head, the notion that it is “highly conserved” is based on an analysis of chemical similarity between residues (10, 17) and should not be taken to imply sequence or structural invariance.

Next-generation sequencing is allowing us to trace with detail the development of highly variable viruses and the antibodies that bind to them. There are no equivalently scalable approaches for determining antigenic development. Improvements in computer hardware and software make MD a credible tool to help bridge that gap. While MM/GBSA may over time be replaced by more self-consistent approaches, this study suggests that it can play a valuable role in binding studies today.

ANCHOR has been identified by other researchers as a tool that can identify key residues in an antigen/antibody complex, and in this work, we find reasonable overall correspondence between key residues identified by ANCHOR and those identified by MM/GBSA. Given that ANCHOR calculations on the two complexes ran in under a minute, the results are impressive, although some highly favorable contacts were not identified. More recent high-speed methods have employed multiscale methods to limit conformational sampling to the region in which a substitution occurs (41). Dourado and Flores (42), for example, restrict conformational sampling to the regions within 12 Å of a substitution. Our results suggest that, in the case of antibody interactions, better results might be obtained by extending sampling to cover the entire span of an epitope, which can exceed 45 Å (36).

## Author Contributions

WL, DM, and AS codesigned the project. WL designed and performed simulations; wrote the manuscript. WL and LS analyzed and interpreted data. WL, LS, DM, and AS reviewed and edited the manuscript.

## Conflict of Interest Statement

The authors declare that the research was conducted in the absence of any commercial or financial relationships that could be construed as a potential conflict of interest.
