# Supplementary material for: Investigating Substitutions in Antibody–Antigen Complexes Using Molecular Dynamics: A Case Study with Broad-spectrum, Influenza A Antibodies
Source: Front Immunol. 2017 Feb 15;8:143. doi: 10.3389/fimmu.2017.00143 (PMC5309259; doi:10.3389/fimmu.2017.00143)
Supplement: Supplementary file 1 [file image_1.pdf]

*Supplementary Material*

# Investigating substitutions in antibody-antigen complexes using molecular dynamics: a case study with broad-spectrum, influenza A antibodies

**William D Lees, Lenka Stejskal, David S Moss, Adrian J Shepherd***

*** Correspondence:** Adrian Shepherd: a.shepherd@mail.cryst.bbk.ac.uk

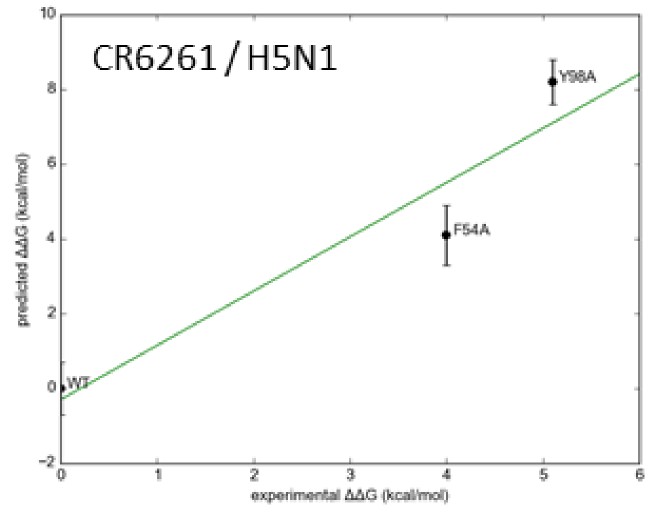
CR6261 / H5N1
Y98A
F54A
WT
predicted ΔΔG (kcal/mol)
experimental ΔΔG (kcal/mol)

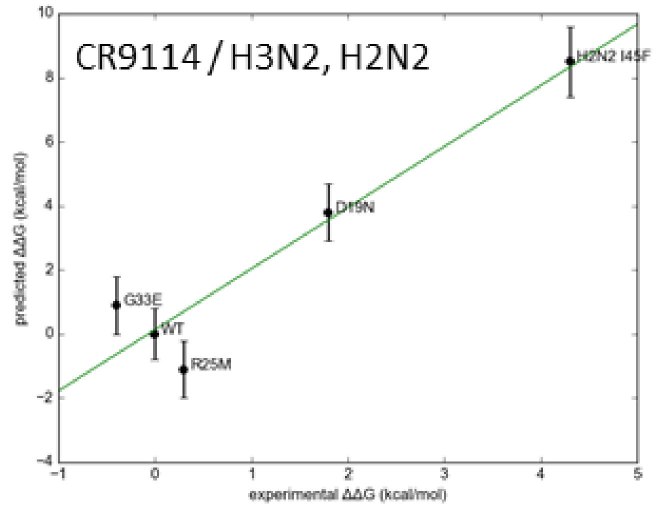
CR9114 / H3N2, H2N2
H2N2 I45F
D19N
G33E
WT
R25M
predicted ΔΔG (kcal/mol)
experimental ΔΔG (kcal/mol)

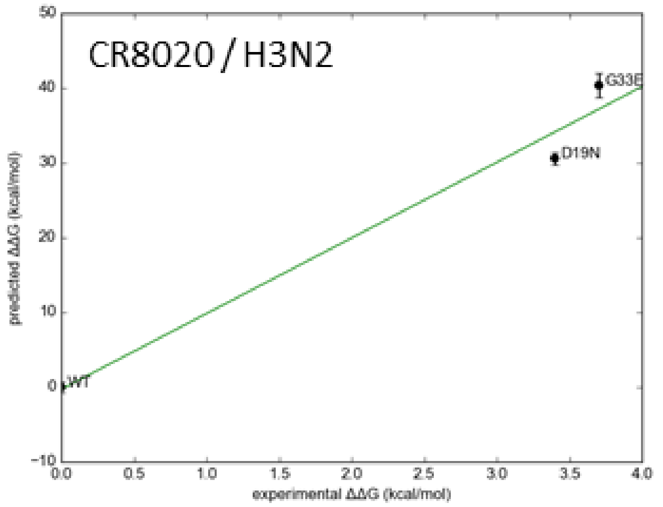
CR8020 / H3N2
G33E
D19N
WT
predicted ΔΔG (kcal/mol)
experimental ΔΔG (kcal/mol)

Figure S1 –Experimentally-derived $\Delta\Delta G_{bind}$ compared with un-scaled predictions
