# Supplementary material for: Investigating Substitutions in Antibody–Antigen Complexes Using Molecular Dynamics: A Case Study with Broad-spectrum, Influenza A Antibodies
Source: Front Immunol. 2017 Feb 15;8:143. doi: 10.3389/fimmu.2017.00143 (PMC5309259; doi:10.3389/fimmu.2017.00143)
Supplement: Supplementary file 2 [file table_1.docx]

Supplementary Material

Investigating substitutions in antibody-antigen complexes using molecular dynamics: a case study with broad-spectrum, influenza A antibodies

William D Lees, Lenka Stejskal, David S Moss, Adrian J Shepherd*

*** Correspondence:** Adrian Shepherd: a.shepherd@mail.cryst.bbk.ac.uk

**Table S1** - List of H1 and H3 strains including the N19 and F45 substitutions

| Subtype | Substitution | Strains |
| --- | --- | --- |
| H1 | I45F | A/Shenzhen/1089/2010  A/Hangzhou/A76/2011  A/Taiwan/90110/2010  A/Guangdong/sz1/2011  A/Guangdong/032/2011  A/Shenzhen/lg63/2011  A/Mexico/InDRE2424/2011  A/Shenzhen/1095/2010  A/Guangdong/147/2011  A/Guangdong/501/2010  A/Mexico/InDRE2425/2011  A/Mexico/InDRE2194/2011  A/Guangdong/379/2010  A/Shenzhen/lg111/2011  A/Hangzhou/3/2009  A/Qingdao/F166/2011  A/HuZhou/01/2010  A/Taiwan/65854/2010  A/Shenzhen/lg2/2011  A/Guangdong/378/2010  A/Hangzhou/A44/2011  A/Zhejiang/NB143/2011  A/Taiwan/3096/2010  A/New Mexico/06/2011  A/Guangdong/4220/2010  A/New Mexico/05/2011  A/Tianjinjinnan/SWL41/2011  A/Hangzhou/B40/2011  A/Shenzhen/ft36/2011  A/Sydney/DD3-27/2010(H1N1)  A/Guangdong/102/2011  A/New Mexico/04/2011  A/California/21/2010  A/Thailand/CU-B4656/2011  A/Mexico/InDRE2419/2011  A/Guangdong/50/2011  A/Guangdong/006/2011  A/Shenzhen/970/2010  A/Guangdong/371/2010  A/Taiwan/90149/2010  A/Tianjinhedong/SWL44/2011  A/Shenzhen/lg3/2011  A/Shenzhen/lg120/2011  A/Hangzhou/A50/2011  A/Guangdong/353/2010  A/Shenzhen/1023/2010  A/New Mexico/07/2011  A/Shandong-Lixia/SWL131/2011  A/Guangdong/067/2011  A/Hangzhou/A61/2011  A/Shenzhen/lg19/2011  A/Guangdong/465/2010  A/Singapore/TT58/2011  A/Guangdong/052/2011  A/Amazonas/129967-IEC/2014  A/Shenzhen/ns06/2011  A/Guangdong/033/2011  A/Guangdong/5303/2010  A/Guangzhou/GIRD74/2010  A/Hangzhou/975/2010  A/Guangdong/379/2010  A/Shenzhen/lg26/2011  A/Hangzhou/B47/2011 |
|  | D19N | A/New Caledonia/09/2004  A/Auckland/30/2006  A/New Caledonia/9/2004  A/Malaysia/643/2003  A/New Caledonia/9/2004  A/Malaysia/687/2003  A/Sydney/65/2005  A/Singapore/GP3667/2010  A/Malaysia/643/2003  A/Sydney/65/2005  A/Malaysia/687/2003  A/Auckland/30/2006  A/Madrid/RC11/2009(H1N1)  A/Taiwan/90236/2011  A/Sri Lanka/21386/2010 |
| H3 | D19N | A/Waikato/1/2004  A/Pavia/07/2014  A/New York/427/1999  A/California/NHRC395507/2013  A/Beijing/353/89 |
|  | N49D | A/New York/752/1993  A/New York/704/1994  A/New York/742/1994  A/New York/743/1994  A/New York/734/1994 |
|  | E325R | A/Yamagata/K74/2006 A/Yamagata/K32/2006 A/Miyagi/S793/2006 A/Fukuoka/F316/2006 A/Miyagi/S709/2006 |
