# Supplementary material for: Investigating Substitutions in Antibody–Antigen Complexes Using Molecular Dynamics: A Case Study with Broad-spectrum, Influenza A Antibodies
Source: Front Immunol. 2017 Feb 15;8:143. doi: 10.3389/fimmu.2017.00143 (PMC5309259; doi:10.3389/fimmu.2017.00143)
Supplement: Supplementary file 3 [file table_2.docx]

Supplementary Material

Investigating substitutions in antibody-antigen complexes using molecular dynamics: a case study with broad-spectrum, influenza A antibodies

William D Lees, Lenka Stejskal, David S Moss, Adrian J Shepherd*

*** Correspondence:** Adrian Shepherd: a.shepherd@mail.cryst.bbk.ac.uk

**Table S2** – Changes in mean distance between the centres of gravity of selected pairs of residues in the CR6261 complex with the substitution HA2 D19N, compared to the WT complex. Distances were computed by *cpptraj* from the final 2ns of the initial production simulation.

| **Residue Pair** | | **distance WT - distance D19N (**Å) | **binding energy in D19 relative to WT** |
| --- | --- | --- | --- |
| K292 | F74 | -0.10 | increased |
| L52 | F74 | 0.52 | increased |
| K292 | D72 | -0.77 | decreased |
| D46 | N31 | 1.52 | increased |
| Q42 | N31 | 1.54 | increased |
| Q42 | N97 | 0.52 | increased |
| N19 | Y100A | -0.35 | decreased |
